# Characterizing the Mechanism of Action of Double-Stranded RNA Activity against Western Corn Rootworm (*Diabrotica virgifera virgifera* LeConte)

**DOI:** 10.1371/journal.pone.0047534

**Published:** 2012-10-11

**Authors:** Renata Bolognesi, Parthasarathy Ramaseshadri, Jerry Anderson, Pamela Bachman, William Clinton, Ronald Flannagan, Oliver Ilagan, Christina Lawrence, Steven Levine, William Moar, Geoffrey Mueller, Jianguo Tan, Joshua Uffman, Elizabeth Wiggins, Gregory Heck, Gerrit Segers

**Affiliations:** 1 Biotechnology Division, Monsanto Company, Chesterfield, Missouri, United States of America; 2 Regulatory Division, Monsanto Company, St. Louis, Missouri, United States of America; University of Kentucky, United States of America

## Abstract

RNA interference (RNAi) has previously been shown to be effective in western corn rootworm (WCR, *Diabrotica virgifera virgifera* LeConte) larvae via oral delivery of synthetic double-stranded RNA (dsRNA) in an artificial diet bioassay, as well as by ingestion of transgenic corn plant tissues engineered to express dsRNA. Although the RNAi machinery components appear to be conserved in Coleopteran insects, the key steps in this process have not been reported for WCR. Here we characterized the sequence of events that result in mortality after ingestion of a dsRNA designed against WCR larvae. We selected the Snf7 ortholog (DvSnf7) as the target mRNA, which encodes an essential protein involved in intracellular trafficking. Our results showed that dsRNAs greater than or equal to approximately 60 base-pairs (bp) are required for biological activity in artificial diet bioassays. Additionally, 240 bp dsRNAs containing a single 21 bp match to the target sequence were also efficacious, whereas 21 bp short interfering (si) RNAs matching the target sequence were not. This result was further investigated in WCR midgut tissues: uptake of 240 bp dsRNA was evident in WCR midgut cells while a 21 bp siRNA was not, supporting the size-activity relationship established in diet bioassays. DvSnf7 suppression was observed in a time-dependent manner with suppression at the mRNA level preceding suppression at the protein level when a 240 bp dsRNA was fed to WCR larvae. DvSnf7 suppression was shown to spread to tissues beyond the midgut within 24 h after dsRNA ingestion. These events (dsRNA uptake, target mRNA and protein suppression, systemic spreading, growth inhibition and eventual mortality) comprise the overall mechanism of action by which DvSnf7 dsRNA affects WCR via oral delivery and provides insights as to how targeted dsRNAs in general are active against insects.

## Introduction

RNA interference (RNAi) is a gene silencing mechanism triggered by double-stranded RNA (dsRNA) [1]. The RNAi pathway is essential for protection against viral infections [2], [3] and for regulation of eukaryotic gene expression. The RNAi pathway has been described and used to study gene function in classical genetic model organisms for over a decade. In insects, multiple studies have confirmed the existence of the RNAi pathway by injection of dsRNAs [4], [5], [6].

More recently, RNAi via ingestion has been suggested as a potential tool for insect control. Several studies have demonstrated that dsRNAs can be successfully fed to insects either through artificial diet or expressed in transgenic host plants, resulting in mortality of the targeted species [7], [8]. Much focus has been on economically important pests, including western corn rootworm (WCR, *Diabrotica virgifera virgifera*), southern corn rootworm (SCR, *Diabrotica undecimpunctata howardii*), Colorado potato beetle (*Leptinotarsa decemlineata*) [7], cotton bollworm (*Helicoverpa armigera*) [9], beet armyworm (*Spodoptera exigua*) [10] and brown planthopper (*Nilaparvata lugens*) [11]. Other recent studies examined the potential to use RNAi to control household/structural pests such as termites [12] and insect vectors of disease such as Tse-tse flies and mosquitos [13], [14]. Once the dsRNA is ingested by these insects, it is thought to be taken up by midgut cells and processed by the native RNAi machinery. The RNAi pathway is initiated by cleavage of dsRNA into short interfering (si)RNAs by the nuclease Dicer [1]. The siRNAs then bind to a complex of proteins known as RNA induced silencing complex (RISC) which leads to specific suppression of the target mRNA. This specific suppression can cause lethality if the target mRNA encodes a protein with an essential function within the insect.

One of the factors that can influence RNAi efficiency in insects is the capacity of cells to uptake dsRNA. A primary route of exposure for dsRNA is oral ingestion, however, ingestion is not synonymous with uptake; the dsRNA must enter cells of the target insect for the dsRNA to interact with the RNAi pathway, resulting in downstream effects [15]. In some organisms, cells have the ability to uptake dsRNA from the extracellular environment and spread the effect to neighboring cells [16], [17]. This process, called non-cell autonomous RNAi, was first described in *C. elegans* [1], where several genes have been implicated in dsRNA uptake and subsequent spreading. There are several models explaining dsRNA entry and initiation of RNAi silencing in different organisms [18], [19], [20], [21]. Systemic RNAi has also been documented in WCR [22] and other insects [23], [24] via dsRNA injection; however the mechanism(s) of dietary uptake of dsRNAs and systemic spreading of RNAi have yet to be fully characterized in an insect model.

Other factors can also influence the efficiency of RNAi in insects, including dsRNA concentration, potency and efficacy against the target, sequence and length of the dsRNA, persistence of gene silencing, and the insect life-stage [7], [8], [25], [26]. In general, long dsRNAs that incorporate a high degree of sequence match to mRNAs in the target insect have greater potential for efficacy as a result of the number of siRNAs that can be produced. [7].

The results described herein characterize the mechanism of action of dsRNA in WCR upon ingestion, including (1) cellular uptake of dsRNA; (2) non-cell autonomous spread and target mRNA and protein suppression and (3) growth inhibition preceding lethality of the WCR larvae. The data also provide insights into the relationship between the time of exposure to dsRNA and concentration effects of dsRNA on WCR larval survival. Taken together, the results presented here provide a more complete understanding of the events that result in mortality from a dietary exposure to a dsRNA targeting an insect mRNA.

The WCR Snf7 ortholog (DvSnf7) is a component of the ESCRT-III complex (endosomal sorting complex required for transport), which is involved in essential biological processes including sorting of cell membrane receptors [27], [28], [29]. Due to its vital cellular function resulting in WCR mortality at relatively low concentrations when targeted by dsRNA [7], DvSnf7 was selected as the target mRNA to investigate the molecular mechanisms leading to WCR mortality after feeding on dsRNA. The data gathered in this study on the overall mechanism of action of DvSnf7 dsRNA by oral delivery to WCR, suggest the possibility of using DvSnf7 as a potential RNAi target for the control of WCR via transgenic approaches.

## Results

### Toxicity of dsRNA against the target organism

**Figure 1:**
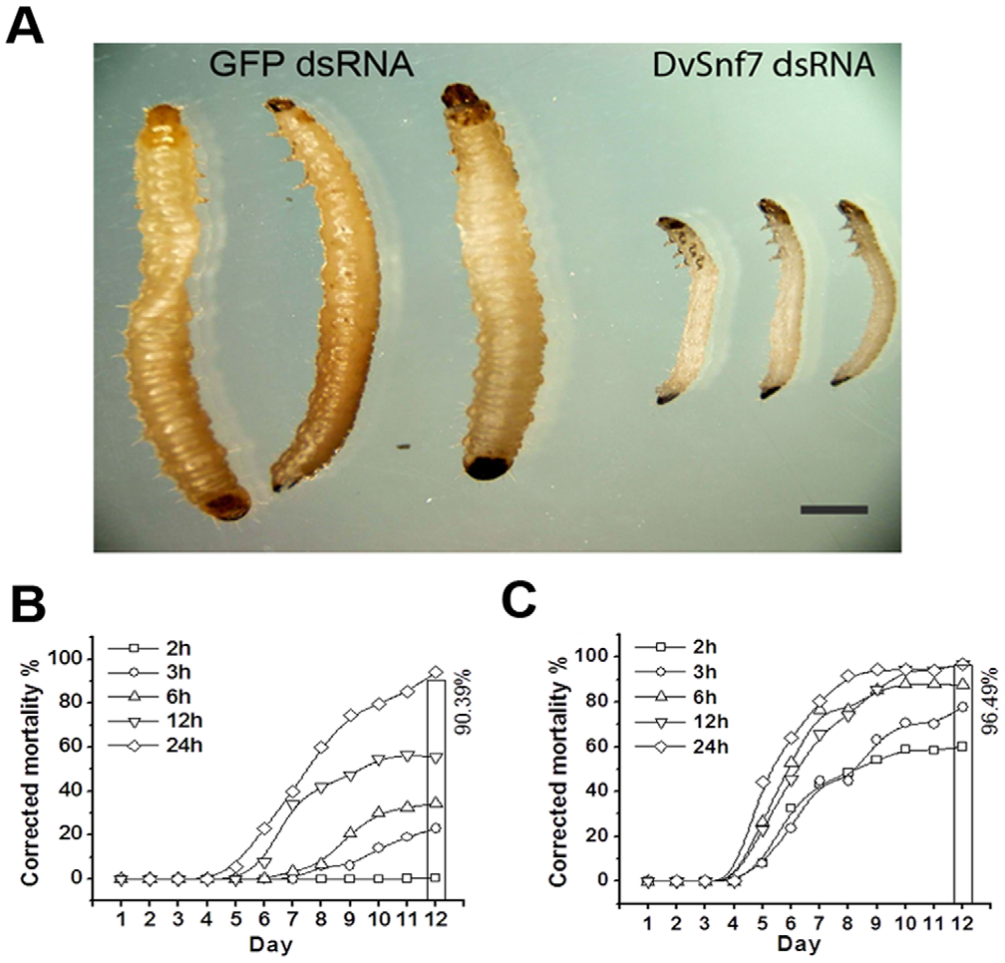
dsRNA DvSnf7 direct toxicity in Western Corn Rootworm (WCR, *Diabrotica virgifera virgifera*) larvae. A) Growth inhibition in second instar larvae fed on diet overlaid with 1000 ng/mL DvSnf7 dsRNA for 5 days compared to larvae fed with control diet containing similar concentration of GFP dsRNA. Scale bar  = 1 mm. Mortality of WCR larvae to varying exposure times of DvSnf7 dsRNA incorporated into diet bioassay at B) 50 ng and C) 1000 ng of dsRNA/mL diet. Open columns represents the mortality of 12-day continuous feeding.

To assess efficacy of an ingested dsRNA against WCR larvae, a 240 bp dsRNA targeting DvSnf7 was fed continuously to second instar larvae at 1 µg dsRNA/mL diet. The larvae fed on DvSnf7 dsRNA showed noticeable growth inhibition 5 days after feeding (Fig. 1A), when compared to control larvae fed Green Fluorescent Protein (GFP) dsRNA. To fully characterize the response of WCR to DvSnf7, time- and concentration–response assays were performed in 12-day artificial diet incorporation bioassays. Additionally, concentration-response assays were conducted with the closely related species SCR, to compare responses and to evaluate the use of SCR as a surrogate for WCR. The SCR 240 bp Snf7 ortholog shares >98% sequence match (data not shown) to WCR 240 bp DvSnf7 dsRNA and in our facility SCR has proven to be more amenable to laboratory bioassays than WCR. Mean LC_50_ values from a combined analysis with results from three independently replicated WCR and SCR differed by three-to-four fold with mean LC_50_ values of 4.3 and 1.2 ng DvSnf7 dsRNA/mL diet, respectively, and comparable slopes for concentration responses (Table 1).

**Table 1:** Western Corn Rootworm (WCR, *Diabrotica virgifera virgifera*) and Southern Corn Rootworm (SCR, *Diabrotica undecimpunctata howardi*) LC_50_ values in 12-day diet incorporation bioassays.

Insect^1^	LC_50_ Values and 95% Confidence Intervals (ng DvSnf7 dsRNA/mL diet)	Slope and 95% Confidence Intervals	Chi-Square for Significance of Concentration Response
WCR	4.3 (2.7–6.2)	3.1 (2.3–3.9)	p<0.0001
SCR	1.2 (0.6–1.9)	3.5 (2.3–4.7)	p<0.0001

1 Replicate assays were performed on three different days with three separate batches of insects.

To characterize the relationship between duration of exposure and levels of effect, WCR larvae were exposed separately to two 240 bp DvSnf7 dsRNA concentrations representing approximately 10 and 225 times the mean 12-day LC_50_ value for WCR (Table 1), and then transferred to untreated diet at specific time points over 12-days. At a concentration of 50 ng DvSnf7 dsRNA/mL diet, a 2 h initial feeding period resulted in no mortality of WCR larvae in the 12 day assay, whereas increasing the feeding duration to 3 h at the same concentration resulted in >20% mortality (Figure 1B). As exposure duration increased, there was a concomitant increase in percent mortality. The longest exposure durations of 12 and 24 h resulted in >50% mortality at 12 days, establishing an inverse relationship between duration of exposure and time required to reach 50% corrected mortality (Figure 1B). In comparison, at the higher concentration of 1000 ng DvSnf7 dsRNA/mL diet, exposure duration and time to reach 50% mortality was considerably shortened, and all exposure durations resulted in >50% WCR mortality (Figure 1C).

### DsRNA size versus activity relationship

To characterize the size-activity relationship of dsRNA against corn rootworm, a dsRNA size series experiment was performed against SCR. A series of 9 different embedded dsRNA sequence lengths (27 bp of DvSnf7 embedded in artificial carrier) and 7 different embedded dsRNA sequence lengths (21 bp of DvSnf7 embedded in artificial carrier) as mentioned in the *Size-activity relationships assays* in methods section were tested to evaluate their biological activity against SCR in 12-day diet bioassays. From these bioassays, a size-activity relationship was established: significant SCR mortality was only detected with sequence lengths ≥60 bp (p>0.05; Table 2). In addition, at a total length of ≥70 bp, SCR mortality was ≥95% at a concentration of 23 ng of DvSnf7 dsRNA/mL diet, representing approximately 20-times the 12-day SCR LC_50_ value. These bioassay results suggest that a size cut-off of approximately 60 bp for a dsRNA is required to achieve significant activity against corn rootworm. A similar level of biological activity against SCR was obtained in separate bioassays with the series of 21 bp embedded in a 240 bp length sequence. All 21 bp sequences embedded in a carrier of total length of 240 bp demonstrated similar activity, whereas ingestion of a 21 bp siRNA (21.3) not embedded in a carrier sequence did not result in significant mortality at the highest concentration tested (p>0.05; Table 3).

**Table 2:** Summary of synthesized dsRNA used to determine the biological activity of different sized molecules at an exposure concentration of 23 ng DvSnf7 dsRNA/mL in 12-day diet bioassays with Southern Corn Rootworm, (SCR, *Diabrotica undecimpunctata howardi*).

dsRNA Tested	Description	Percent Mortality^1, 2^
27	27 bp without carrier	0
27_40	27 bp in neutral carrier to 40 bp	0
27_50	27 bp in neutral carrier to 50 bp	16
27_60	27 bp in neutral carrier to 60 bp	68*
27_70	27 bp in neutral carrier to 70 bp	95*
27_80	27 bp in neutral carrier to 80 bp	95*
27_90	27 bp in neutral carrier to 90 bp	95*
27_100	27 bp in neutral carrier to 100 bp	95*
27_150	27 bp in neutral carrier to 150 bp	96*
27_240	27 bp in neutral carrier to 240 bp	95*
240 bp	DvSnf7 full 240 bp, no carrier	95*
240 Filler	Neutral carrier sequence, 240 bp	0

1 Mortality was corrected using Abbott's formula.

2 Values with an asterisk were determined to have significantly higher mortality compared to the control (water-only) with a one-sided Fisher's Exact Test (p>0.05).

**Table 3:** Comparison of LC_50_ values for siRNA and dsRNAs in 12 day Southern Corn Rootworm, (SCR, *Diabrotica undecimpunctata howardi*) diet bioassays.

dsRNA Tested^1^	Description	LC_50_ Values (ng/mL)^2^
21.3	21 bp 21.3 without carrier	>100^3^
21.1	21.1 bp in neutral carrier to 240 bp	13.8 (10.4–18.7)^4^
21.2	21.2 bp in neutral carrier to 240 bp	15.1 (10.9–23.1)
21.3	21.3 bp in neutral carrier to 240 bp	16.9 (9.40–23.00)
21.4	21.4 bp in neutral carrier to 240 bp	20.3 (15.6–29.3)
21.5	21.5 bp in neutral carrier to 240 bp	14.5 (9.7–25.1)
21.6	21.6 bp in neutral carrier to 240 bp	13.8 (8.9–26.4)
21.7	21.7 bp in neutral carrier to 240 bp	8.0 (4.7–14.6)
240	DvSnf7 full 240 bp, no carrier	1.2 (0.5–2.7)

1 See supplemental materials for individual sequences.

2 Slopes for each of the dsRNAs tested were not significantly different (p>0.05) with a shared slope ± standard error of 2.38±0.22. All concentration reponse curves showed a significant concentration-effect relationship p<0.001.

3 A concentration response was not observed.

4 Diet concentrations bracketing the LC_50_ value are provided as an estimate of the 95% confidence intervals.

### Uptake of dsRNA into WCR midgut cells

**Figure 2:**
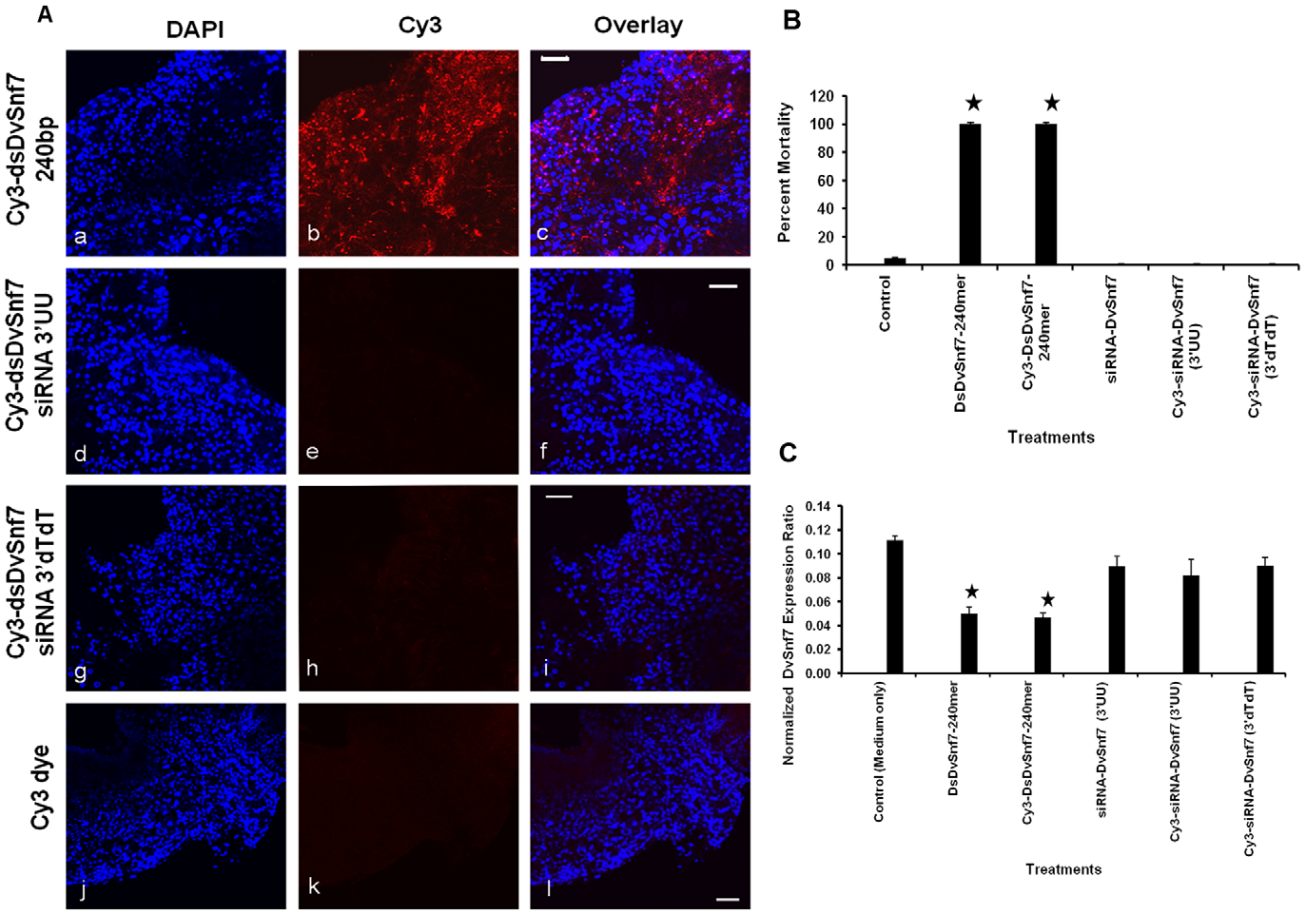
Uptake of dsRNA by Western Corn Rootworm (WCR, *Diabrotica virgifera virgifera*) by midgut cells. (A) Cy3-labeled 240 bp DvSnf7 dsRNA is taken up by WCR midgut cells (b–c), while DvSnf7 siRNAs are not (e–f, h–i). Nuclei DAPI staining was used to visualize midgut cells (a, d, g, j). Controls with Cy-3 dye alone do not show intracellular incorporation (k–l); Scale Bar: 50 µm.(B) Diet bioassays confirmed that labeled 240 bp dsRNA retains activity, while siRNAs are inactive. Percent mortality was determined 12 days after continuous exposure of WCR neonates to 100 ng/mL of dsRNA/siRNA. (C) Real-time RT-PCR of midgut tissue cultures exposed to 1 µg/100 µL of insect medium of dsRNA/siRNA. Snf7 mRNA levels are reduced after exposure to DvSnf7 240 bp dsRNA, while no mRNA reduction is observed after tissue incubation with siRNAs or control medium. Stars represent values significantly different from controls (p = 0.05; t-test).

A WCR midgut tissue culture was developed to evaluate whether dsRNA and siRNAs were effectively taken up by insect midgut cells. 240 bp DvSnf7 dsRNA and DvSnf7 siRNA (21.3, Table 3) were labeled with Cy3 dye to allow for microscopic visualization. The labeled molecules were then incubated with WCR midgut tissue culture. The 240 bp Cy3-dsRNA was localized inside the cells, while Cy3-siRNAs were not (Fig. 2A). Controls with insect medium containing unincorporated Cy3 dye showed no fluorescence (Fig. 2A). Additional controls with unlabeled dsRNA and siRNA molecules co-incubated with unincorporated Cy3, as well as insect medium, showed no fluorescence (data not shown). Both 240 bp dsRNA and siRNAs were used in WCR diet overlay bioassays (at 100 ng/mL diet) to confirm that Cy3–labeling did not interfere with the RNAi response. There was no difference in activity between unlabeled and Cy3-labeled 240 bp dsRNAs (Fig. 2B). Additionally, DvSnf7 mRNA levels were measured in midgut tissues exposed to both Cy3-labeled and unlabeled dsRNA/siRNA for 24 h by real-time PCR. DvSnf7 mRNA levels were suppressed 2-fold only in midguts exposed to labeled/unlabeled 240 bp dsRNA but not in midguts exposed to labeled/unlabeled siRNA (Fig. 2C), confirming that the 240 bp dsRNA is still active upon cellular uptake. These results suggest that a size selection mechanism is present at the cellular uptake level, allowing for the effective uptake of relatively long dsRNA molecules and excludes siRNAs.

### Target suppression and systemic RNAi effect in WCR

**Figure 3:**
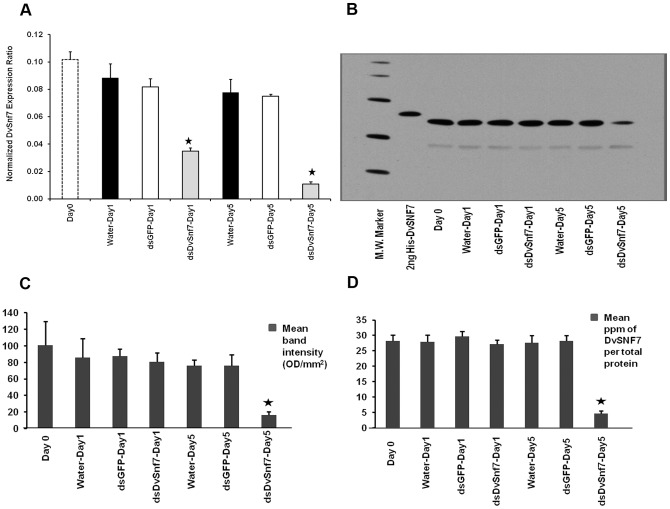
DvSnf7 mRNA and DvSNF7 protein suppression by RNAi. DvSnf7 dsRNA causes suppression at mRNA (A) and protein (B–D) levels in Western Corn Rootworm (WCR, *Diabrotica virgifera virgifera*). (A) Real-time RT-PCR results showing significant decrease in DvSnf7 mRNA expression in insects fed with 60 ng/mL of DvSnf7 240 bp dsRNA continuously for one and five days. Insects fed with control diets containing water or GFP dsRNA do not show mRNA suppression. (B) Western blot results using anti-SNF7 antibody showed DvSNF7 protein suppression in insects fed with DvSnf7 240 bp dsRNA after 5 days, which was confirmed by quantification of the Western blot by densitometry (C) as well as ELISA (D). Stars represent values significantly different from controls (p = 0.05; t-test).

To verify the efficiency of target suppression, DvSnf7 mRNA levels were measured by real-time RT-PCR. DvSnf7 transcript levels were significantly reduced as early as one day after DvSnf7 dsRNA feeding, and target suppression was even more pronounced in insects feeding on DvSnf7 dsRNA for five days (Figure 3A). To evaluate if DvSNF7 protein levels also decreased, an anti-SNF7 antibody was generated and used to detect DvSNF7 protein in these larval samples by Western blot and ELISA. Immunoprecipitation of DvSNF7 protein from a WCR protein extract followed by mass spectrophotometry confirmed the specificity of the anti-SNF7 antibody (data not shown). Fig. 3B–D shows that DvSNF7 protein levels were not altered in larvae feeding on DvSnf7 dsRNA for 1 day of exposure. However, DvSNF7 protein levels were significantly reduced in larvae after 5 days of feeding on DvSnf7 dsRNA.

**Figure 4:**
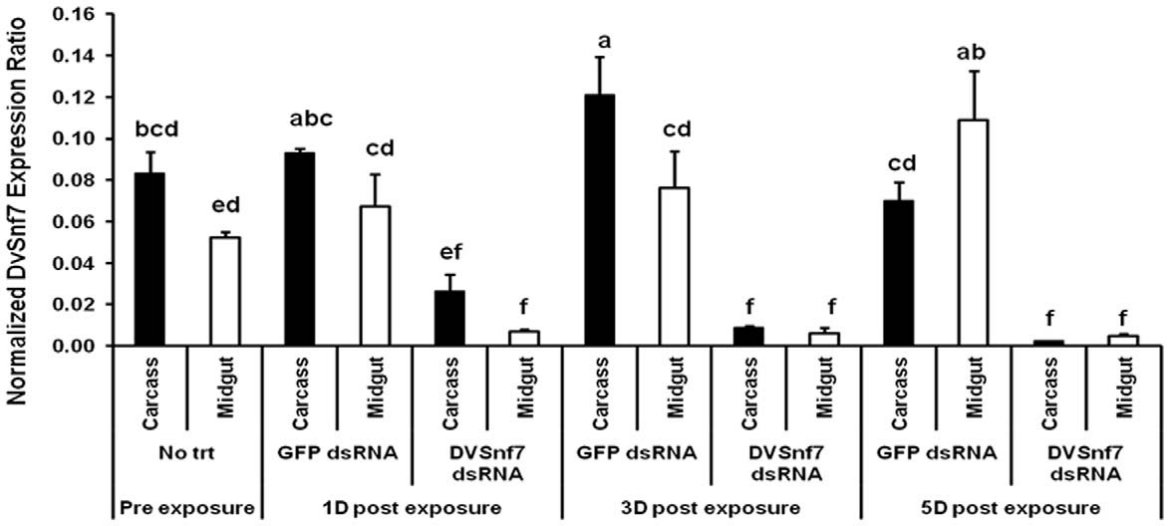
RNAi effect spreads to other tissues beyond the midgut in Western Corn Rootworm (WCR, *Diabrotica virgifera virgifera*). DvSnf7 mRNA levels were decreased in isolated midgut and carcass tissues of second instar larvae fed for 24 hours with 1000 ng/mL diet of DvSnf7 240 bp dsRNA, as assessed by Real-time RT-PCR. Further mRNA suppression is observed in the carcass and midgut at days 3 and 5 post-feeding. Means followed by same letter are not significantly different (p = 0.05; t-test).

To test whether the DvSnf7 suppression effect is systemic or limited to the midgut (the primary tissue in contact with dsRNA via feeding), insect midgut and carcass tissues were isolated and DvSnf7 mRNA levels were measured by real-time RT-PCR. Results showed that DvSnf7 mRNA levels were high in both tissues of pre-exposure treatment and GFP dsRNA fed larvae, and were statistically significant from the levels in the counterpart tissues of DvSnf7 dsRNA fed larvae (Fig. 4). DvSnf7 mRNA levels reduced in both midgut and carcass as early as one day after ingestion of DvSnf7 dsRNA, although suppression was more pronounced in the midgut (10-fold) than in the carcass (4-fold). Suppression was almost similar in both tissues by the third day post-feeding (13-fold) and it increased in the carcass tissue (29-fold) when compared to the midgut tissue (22-fold) by fifth day post-feeding (Fig. 4). These results indicate that the RNAi response acts non-cell autonomously, spreading from the midgut to other WCR tissues.

## Discussion

The RNAi pathway is a well conserved mechanism in insects [30] that holds high potential as an insect control technology, as many insects have been found to be susceptible to orally ingested dsRNA. Although the ability to successfully achieve efficacy with dsRNA via oral ingestion appears wide-spread for insects, it is not universal and there is a wide range in sensitivities across taxa [31].

Huvenne and Smagghe [25] listed multiple factors influencing the efficiency of RNAi, including concentration, sequence, and length of the dsRNA. Our data provide empirical support for the suggestions made in Huvenne and Smagghe [25] on several fronts. We have demonstrated a clear concentration–response in two *Diabrotica* species, WCR and SCR (Coleoptera: Chrysomelidae) that share 98% sequence identity of the 240 bp dsRNA targeted to the Snf7 ortholog, and exhibited comparable sensitivity. The relatively small increase in sensitivity of SCR to DvSnf7 dsRNA compared to WCR is similar to our observations with Bt proteins such as Cry3Bb1 (JU, personal observation) which may reflect overall physiological differences such as food consumption and relative growth rate (SL, personal observation). Not only are sequence identity and concentration determinants of dsRNA efficiency, but also the temporal nature of exposure. For example, a concentration of 1000 ng DvSnf7 dsRNA/mL diet resulted in an earlier onset of lethality and greater overall WCR toxicity compared to a concentration of 50 ng DvSnf7 dsRNA/mL diet. After 2 h of exposure, there was >50% WCR mortality at 1000 ng DvSnf7 dsRNA/mL diet, whereas at 50 ng DvSnf7 dsRNA/mL diet a 12 h exposure was required to reach >50% mortality. These data indicate that only a short duration of feeding on dsRNA targeted to a vital cellular function may be required to achieve a high degree of efficacy in WCR and this result is consistent with the high level of Snf7 mRNA suppression observed after one day of exposure.

Induction of RNAi via an oral route of exposure requires efficient uptake of dsRNAs by midgut cells followed by suppression of the target mRNA leading to significant effects on growth, development and survival. Studies utilizing fluorescently labeled dsRNA as a means of investigating uptake in insects are currently lacking, and have been recommended in a recent review on RNAi in insects for pest control purposes [32]. This investigation utilized fluorescently-labeled dsRNA to examine for the first time the uptake of a specific dsRNA by WCR. Our WCR tissue culture experiments demonstrated effective uptake of the 240 bp dsRNA along with suppression of the intended target mRNA. They also confirmed the lack of activity of siRNAs against WCR by demonstrating that 21 bp siRNAs are not taken up into midgut cells, suggesting that the dsRNA length plays an essential role in the effectiveness of the RNAi response in WCR larvae. These results were complemented with the evaluation of the dsRNA size-activity relationship. siRNA/dsRNA molecules of 21 and 27 bps were not effective against corn rootworm larvae in diet bioassays. In addition, ingested dsRNAs ≥60 bp, containing an active 27 bp sequence, were shown to be efficacious in 12-day bioassays against SCR, while dsRNAs <60 bp did not result in relatively high mortality in 12-day bioassays. This absence of activity for dsRNAs <60 bps is not attributed to a lack of stability in the insect diet. Borgio & Upadhyay et al. [33], [34] demonstrated that 21 bp siRNAs are stable in an insect diet for seven days and we have confirmed stability of the DvSnf7 dsRNA in SCR diet when evaluated for time periods up to 14 days (data not reported). These experiments also showed that a 240 bp dsRNA with 100% complementarity to the target mRNA is more efficacious compared to the same length dsRNA with only 21 bp of sequence match to the target WCR mRNA, and is at least three orders of magnitude more toxic than Bt Cry3Bb1 against WCR [35]. This could be due to the fact that the processing of long dsRNAs with 100% match with the target mRNA by the RNAi machinery will result in multiple target-specific siRNAs which would provide a greater number of siRNAs available to cause target mRNA suppression and increase mortality. Additionally, because a 240 bp dsRNA would produce 100′s of siRNA's, the likelihood of rootworm developing resistance to this molecule via SNP's in the target DvSnf7 mRNA, is greatly reduced [36].

Data from *Drosophila* S2 cells support our results; long dsRNAs are efficiently taken up but siRNAs are only taken up with the aid of transfection reagents [19]. In contrast, Kumar et al. [37] reported efficient suppression of acetylcholinesterase in the *Helicoverpa armigera* via incorporation of siRNAs in diet, however, this study did not report on the effect of dsRNA. Not all insect orders exhibit the same response to orally ingested dsRNAs [25]. Lepidopteran responses to either injected or oral dsRNA delivery are highly variable, which may indicate that Lepidoptera possess a high degree of plasticity (high variation among species) in their uptake mechanisms and RNAi response [15], [33]. Additionally, specific gene silencing by siRNA treatment was observed via oral route in sucking pests such as white flies [33]. Hence, the differences in uptake selectivity seen between our studies and those of Kumar et al. [37] and Upadhayay et al. [33] may be a factor of the test species used as well as the gene target. Although we have demonstrated that long dsRNAs are successfully taken up by WCR midgut cells and siRNAs are not, the mechanism of uptake at the cellular level is still unknown. Further molecular and bioinformatics studies are required to understand whether dsRNA uptake occurs via SID-like receptors, receptor-mediated endocytosis, an unknown mechanism, or a combination of uptake mechanisms.

Once dsRNA has crossed the cell membrane, it is processed by the RNAi machinery ultimately leading to mRNA degradation. Bioassays with WCR exposed to a DvSnf7 dsRNA concentration approximating the 12-day LC_99_ in WCR diet bioassays showed that DvSnf7 transcript levels started to decrease 1 day after neonate larvae started feeding on diet containing dsRNA, and by the fifth day the suppression was greater than 80%. Although DvSNF7 protein levels were steady 1 day after feeding, they were significantly reduced (80% decrease) after 5 days compared to pre-exposure levels. Because mortality started to occur in WCR after 5 days, it was not feasible to collect sufficient tissue for later time points to assess whether complete suppression occurred. Nevertheless, because SNF7 is involved in many essential biological functions, perturbation in the level of this protein is likely enough to result in a cascade of events leading to mortality. A recent publication examining vATPase subunit A mRNA knockdown in adult WCR demonstrates a similar pattern in protein suppression [38]. Specifically, Rangasamy & Siegfried [38] showed that mRNA levels decreased within 24 hours of ingestion of a dsRNA targeting WCR vATPase, while protein levels decreased only after three days of feeding. Even though complete suppression of the vATPase protein was not achieved, the RNAi effect still resulted in mortality.

Although systemic RNAi has been previously described for insects of different orders via dsRNA injection [22], [23], [24] or ingestion [39], [40], this is the first report of systemic RNAi effect via ingestion of dsRNA in a Coleopteran insect. By quantifying mRNA levels in isolated tissues such as midgut and carcass, we were able to demonstrate the RNAi effect in WCR as systemic which agrees with the requirement of longer exposure of relatively low concentrations of DvSnf7 dsRNA to cause demonstrable toxicity. Significant target suppression was obtained in both midgut and carcass tissues after 24 hours. In addition, DvSnf7 mRNA levels decreased faster in the midgut than in the carcass. This is not surprising as the midgut is one of the first tissues that come into contact with the dsRNA upon ingestion. Hence, because the silencing signal is exported from intestinal cells to other cells (in the carcass for example) resulting in target suppression in distant cells, it may likely be associated with a higher likelihood in RNAi efficacy leading to insect death.

By combining the data from the diet bioassays, molecular studies and tissue culture assays, a profile of the course of events that leads to mortality in WCR upon ingestion of a dsRNA emerges. Significant suppression of DvSnf7 mRNA in midgut and carcass tissues was evident 1 day after exposure to the long DvSnf7 dsRNA, though DvSNF7 protein levels were unaffected and no mortality was observed. However three days after the initiation of feeding, DvSnf7 mRNA suppression stabilized in both tissues and continued to spread more in the carcass tissues by five days post exposure. Concomitantly, DvSNF7 protein levels were significantly reduced by five days of expusre to DvSnf7 dsRNA. The timing of suppression and systemic spreading coincides with the onset of mortality in WCR larvae as observed in the diet bioassays. Therefore the general mechanism of action that leads to WCR death upon DvSnf7 dsRNA ingestion includes dsRNA uptake, target mRNA/protein suppression and systemic spread. This work also characterizes the relationship between dsRNA size in regards to uptake and efficacy. In addition, the results presented here, although obtained with a specific mRNA target (DvSnf7), suggests extrapolation to other lethal targets due to the conserved nature of the RNAi machinery. Further studies are underway that will reveal the unique cellular and physiological defects that lead to WCR death upon suppression of the DvSnf7 ortholog [41].

## Materials and Methods

### Insects

For all bioassays, WCR and SCR eggs were received from Crop Characteristics (Farmington, MN). Upon receipt, eggs were maintained at a target temperature of 10°C to 25°C depending on desired hatch time prior to disinfection. Near-hatching eggs were washed and dispensed into plastic containers prior to hatching. Newly hatched neonates (<30 hours post hatch) or second instar larvae were used in all bioassays.

### WCR bioassays

Bioassays were performed using either diet-overlay or diet incorporation methodology. For diet-overlay, WCR diet was prepared according to manufacturer's guidelines for SCR diet (Bio-Serv, Frenchtown, NJ) with a few adjustments, including the addition of Formalin at 0.06% (v/v), 10% KOH (v/v) to increase pH to 9, and lyophilized corn root tissue at 0.62% (w/v). 200 µl of molten diet was pipetted into 24 wells of 96 well plates (Falcon), allowed to solidify at room temperature and 20 µL's of dsRNA solution or water control was overlaid in each well. Plates were air dried and one larvae (<30 hours post hatch) was added per well. Plates were sealed with mylar, ventilation holes added to each well with a #1 or #2 insect pin, and plates incubated at 27°C for 12 days. For diet incorporation assays, dsRNA treatments were prepared by mixing a dosing solution and purified RNAse free water with Bio Serv diet described above by vortex-mixing until homogeneous. A repeat pipettor was used to aliquot 1 mL of diet into individual wells of 48-well plates (Falcon). Plates were allowed to air dry and one larvae (<30 hours post hatch) was added per well with a target number of 72 larvae used for control treatments in each assay, and a target number of 24 larvae for each dsRNA treatment replicate. Plates were sealed with mylar and incubated at 27°C for 12 days, unless otherwise stated.

### Growth inhibition and dsRNA uptake

Diet bioassays for growth inhibition and uptake studies were conducted with WCR. For the growth inhibition study, 10 WCR larvae were fed a single concentration of 1000 ng dsRNA/mL diet in a diet overlay bioassay for a period of 5 days. Growth inhibition was determined against an equal number of WCR larvae fed GFP dsRNA as a control. For the uptake studies, WCR larvae were fed Cy-3 labeled dsRNA DvSnf7 240 bp and the siRNA (21.3) at 100 ng dsRNA/mL diet for 12 days. Control and treatment groups contained a target number of 24 larvae each.

### Concentration-Response Relationship Bioassays

A series of diet incorporation bioassays were conducted to characterize concentration-response relationships and estimate LC_50_ values for dsRNA DvSnf7 against WCR and SCR. Three replicates over time were conducted for each species. For each replicate assay, approximately 24 larvae were infested at each concentration and 72 larvae in negative control. Each bioassay consisted of a buffer control and a geometric series of concentration levels to estimate LC_50_ values. RNAse-free water was used as a control and individual bioassays were deemed acceptable if control mortality was less than 20%.

### Exposure duration bioassays

WCR neonate larvae were fed 50 ng or 1000 ng of DvSnf7 dsRNA/mL diet for 2, 3, 6, 12 and 24 hours followed by exposure to control diet for a total duration of 12 days. Additionally, separate WCR neonates were exposed continuously to the same concentrations for the entire 12 day assay period using artificial diet as described above. In order to expose a large number of larvae, and facilitate removal of subsamples at different time points, Petri dishes (100 mm) were used as exposure chambers. For each treatment concentration, 20 mL of diet was added to each Petri dish and a target number of 100 newly-hatched WCR larvae were added in each dish with a fine haired paintbrush. At each sampling time, 30 to 36 WCR larvae were removed from each DvSnf7 treatment and placed onto 250 µL of untreated diet in 48-well plates. For continuous exposure treatments, larvae were directly added onto diets containing DvSnf7 at 50 or 1000 ng dsRNA/mL diet in 48-well plates. Mortality was recorded daily for the duration of the assay.

### Size-Activity Relationship Studies

Ten dsRNA fragment lengths that contained an active 27 base pair (bp) dsRNA sequence were incorporated into inert carrier sequences with total lengths of 27 (no additional carrier): 40, 50, 60, 70, 80, 90, 100, 150, and 240 bp, and tested against SCR in diet incorporation bioassays (see File S1). The 27 bp size fragment was chosen over a smaller 21 bp fragment due to its ability to provide a better substrate for the dicer enzyme, allowing for efficient cleaving of dsRNA into siRNAs [42]. Additionally, there is 100% sequence match for this 27 bp fragment between WCR and SCR. This 27 bp segment was chosen based upon demonstrated high efficacy against WCR when included as part of longer dsRNAs (data not shown), The chosen 27 bp fragment can be diced into seven different 21 bp fragments (see File S1). In order to compare the activity of the individual 21 bp fragments comprising the 27 bp fragment, each of the seven synthetic 21 bp were also embedded into an inert carrier sequence for a total of 240 bp and fed to SCR in 12-day artificial diet bioassays. Details of dsRNA synthesis method are described in the *dsRNA synthesis* session below.

The inert (neutral) carrier sequence was designed according to strict criteria, including: (1) a standard sequence selection process for inverted repeat transformation constructs (i.e. no 21-mer matches to corn or human), and ideally even fewer matches to WCR sequences (e.g., no 18 out of 21-mer matches or even more stringent); (2) little homology to corn or WCR sequence; (3) similar GC content as the original DvSnf7 240 bp; and (4) little coding capacity (no ATGs in beginning of the sequence, no more than 20 potential amino acids in all six-frames).

SCR larvae for the size series assays were continuously exposed at 23 ng DvSnf7 dsRNA/mL diet for each dsRNA fragment length. Controls for each assay included negative water-only treatments as well as the neutral carrier sequence at 23 ng/mL. To compare the activity of the seven different 21 bp fragments, each of the seven synthetic 21 bp were tested in concentration-response assays with concentrations ranging from 0.24 to 23 ng DvSnf7 dsRNA/mL diet. Control and treatment groups contained a target number of 72 and 24 larvae, respectively, and mortality was evaluated after 12 days. Positive control for the assays was the DvSnf7 240 bp construct. Diet used in these studies was standard SCR diet (Bio-Serve, Frenchtown, NJ) prepared according to the manufacturers guidelines. Dispensing of diet and infesting of SCR larvae was performed as described for WCR above. Bioassay trays were incubated at a target temp of 27°C and 70% relative humidity in complete darkness.

### Snf7 mRNA and protein suppression studies

Diet incorporation bioassays were conducted to expose WCR larvae (≤30 hours old) to the 240 bp DvSnf7 dsRNA to generate tissues for Western Blot, ELISA and real-time RT-PCR analysis. Larvae were exposed to dsRNA continuously and sampled after 0 days (no exposure), one day, and five days. The exposure procedure was followed as described under *Exposure Duration Bioassays* section. To ensure toxicity of DvSnf7 to WCR, 60 ng/mL of dsRNA was incorporated into the diet. This value represents the 12-day LC_99_ for DvSnf7 against WCR as determined by concentration-response bioassays (data not shown). Control treatments of GFP dsRNA and water were performed concurrently with DvSnf7 treatment. Tissue samples were collected and held at −80°C until processed and analyzed for DvSnf7 mRNA and protein suppression.

### Systemic spread studies

Diet-overlay bioassays were conducted to expose 2^nd^ instar WCR larvae to the 240 bp DvSnf7 dsRNA and dissect midgut and carcass tissues for real-time RT-PCR analysis. Larvae were exposed to dsRNA continuously and sampled after 0 days (no exposure), one day, three days and five days. The exposure procedure was followed as described under *Exposure Duration Bioassays* section. Because a higher concentration of dsRNA is required for 2^nd^ instar larvae compared to first instar larvae (data not shown), 1000 ng dsRNA/mL diet was overlaid onto diet. A control treatment with GFP dsRNA was performed concurrently with DvSnf7. Isolated tissue samples were collected and held at −80°C until processed and analyzed for DvSnf7 mRNA.

### dsRNA synthesis

The WCR DvSnf7 target cDNA (Supplementary Information S1) was amplified out of a WCR neonate cDNA library prepared using SuperScript™ First-Strand Synthesis System (Invitrogen), using total RNA extracted with TRIzol reagent (Invitrogen) as template. Primers containing a T7 polymerase promoter region (TAATACGACTCACTATAGGG) at the 5′ end were used to amplify the DvSnf7 240 bp region by PCR (Supplementary Information Table S1). PCR products were cloned into pUC19 (New England Biolabs) between *Eco*RI and *Hind*III restriction sites and sequences confirmed. Plasmid DNA was linearized using *Hind*III restriction enzyme and used as template for dsRNA synthesis using the MEGAscript kit (Ambion), according to the manufacturer's protocol.

GFP target (Supplementary Information S1) cloned into pBTA2 was amplified using gene specific primers (Supplementary Information Table S1) containing the T7 polymerase recognition region at the 5′ end. The PCR product was used as template to synthesize dsRNA with the MEGAscript kit (Ambion) following the manufacturer's protocol.

To investigate the effect of dsRNA length on WCR activity, a 27 bp sequence from WCR DvSnf7 (TAGATGGAACCCTTACAACTATTGAAA) was embedded into an artificial sequence (filler) (Supplementary Information S1), resulting in a 240 bp product. Various dsRNA sizes were made by successively paring down each end of the artificial sequence to create a range of dsRNA sizes (40, 50, 60, 70, 80, 90, 100 and 150 bp) in which the DvSnf7 27 bp sequence was located in the middle of each dsRNA. All seven possible 21 bp fragments from the 27 bp sequence were synthesized and embedded into the inert sequence to form 240 bp fragments. Nucleotides adjacent to the 21 bp that matched the DvSnf7 sequence (including G-U pairings) were mismatched to prevent production of an embedded 22 bp. DNA fragments of the desired sequences containing a single T7 polymerase promoter region (TAATACGACTCACTATAG GG) were cloned into pUC19 (New England Biolabs) between EcoRI and HindIII restriction sites and their sequences confirmed. Two separate clones with the same target region in sense or antisense orientations were used as template for dsRNA synthesis using the MEGAscript kit (Ambion), according to manufacturer's protocol.

To ensure the quality and integrity for the characterization of the dsRNA samples, the following criteria were followed: 1) DNA templates for *in vitro* transcription reactions of dsRNA test materials contained the expected nucleotide sequences, confirmed via DNA sequencing and alignment; 2) dsRNAs had near 100% purity, based on spectrophotometric analysis, in addition to agarose gel results; 3) individual dsRNA fragment lengths matched the expected lengths, confirmed via agarose gel electrophoresis; 4) the final concentration of each dsRNA material was determined via spectrophotometric analysis.

### Labeling of dsRNA/siRNA

One of the seven possible siRNAs (21 bp; Supplementary Information S1) within the 27 bp dsRNA, 21.3 was selected based on the criteria described in [43]. The siRNA 3′ end was modified to dTdT to inhibit nuclease activity during midgut incubation in tissue culture medium. Cy-3 labeled and unlabeled versions of these siRNAs were purchased from Sigma. Cy3-labeling of 240 bp DvSnf7 dsRNA was performed using the Silencer siRNA labeling kit (Ambion), according to manufacture's instructions. Adequate labeling of dsRNA was confirmed by loading both labeled and unlabeled dsRNAs onto a 1% agarose gel. The gel was stained with 1 µg/mL ethidium bromide in 1X TBE (Roche). Bands were visualized using a UV transilluminator (Bio-Rad).

### Midgut tissue culture assays

Second-instar WCR larvae grown on artificial diet were transferred to 24-well plates containing 1.5 cm filter paper circles wetted with sterile 1X PBS (Phosphate buffered saline (Roche) +0.5% gentamicin (Sigma) +0.1% 100X antibiotic-antimycotic solution (Sigma)) for 2–3 h. This step was included to minimize the presence of gut contents. Larvae were surface sterilized by immersion in successive 2-min washes of 70% ethanol and 0.1% Clorox solution, and washed twice in sterile 1X PBS. Midguts were dissected under aseptic conditions in a horizontal laminar airflow work station in sterile 1X PBS. Midguts were rinsed in sterile 1X PBS twice to eliminate gut contents, then washed in sterile 1X PBS followed by sterile Insect 420 medium (Sigma) at 1∶3 ratio, and finally into sterile Insect 420 medium (with 100 µg/mL gentamicin and 1X antibiotic-antimycotic solution (Sigma)). Midguts were subsequently incubated in batches of 3–5 per well in 96-well plates containing 100 µL sterile insect medium for 4 h at 25°C. Labeled and unlabeled dsRNA and siRNAs were added at 1 µg/100 µL insect medium and incubated for 15 h at 25°C protected from direct light. Unincorporated Cy3 dye was used as a control at 1∶100 in insect medium. The entire experiment was performed in triplicate containing 3–5 midguts per replicate. Midguts were retrieved from culture plates, washed twice with 1X PBS and fixed in 4% Paraformaldehyde for 1 h at room temp. Midguts were washed three times with 1X PBST (PBS +0.1% Tween) for 5 minutes each and counterstained with DAPI (Sigma, 10 mg/mL of stock diluted to 1∶1000) for 5 min. Finally, midguts were washed once in 1X PBST and mounted onto a glass slide containing Slow fade antifade solution (Invitrogen). Images were captured using a 550 nm (Cy3) and 360 nm (DAPI) laser for excitation and 570 nm (Cy3) and 450–460 nm (DAPI) for emission filter sets, under a confocal microscope. Scanned images were processed using LSM (Carl Zeiss AIM; version 4.2) and Adobe Photoshop (CS5 software; version 12.0×32) software.

### Real-time RT-PCR

RNA was extracted from insect tissues using TRIzol reagent (Invitrogen) following manufacturers' instructions. RNA was quantified using a spectrophotometer (Nanodrop), diluted to 50 ng in RNAse free water and used as template for real-time RT-PCR using the CFX manager software (Biorad), according to manufacture's instructions. Reactions included 1 µL RNA (50 ng/µL), 6.25 µL of SYBR Green mix (*Iscript one-step RT-PCR* kit, BioRad), 0.5 µL 10 pmol forward and reverse primers, 0.25 µL reverse transcriptase (BioRad) and 4.0 µL RNAse free water, for a total volume of 12 µL. Reactions were set-up in 96-well Microseal PCR plates (Biorad) in triplicate. Appropriate controls including no-template control (NTC) and no reverse transcriptase control (NRT) were included. Tubulin (endogenous control; reference gene, Supplementary Information S1) primers were used simultaneously for normalization. Standard curves were generated using known concentrations of DvSnf7 plasmid clone DNA. Relative expression levels were determined using the standard curve.

### Western blots and ELISA

Full length His-tagged DvSNF7 protein was produced in *E. coli* and the N-terminal His-tagged protein was purified by sequential Ni-NTA and size exclusion chromatography. Concentrations were determined by amino-acid analysis. Purity was >95% and identity was confirmed by mass spectrophotometry. This sample was used for antibody generation and as protein standard in western blots and ELISA. Polyclonal antibodies were raised in New Zealand White rabbits against the N-terminal His-tagged full length DvSNF7 protein. IgG was Protein A (Bio-Rad) affinity-purified from sera and biotinylated (Thermo) for use as detection antibody in ELISA experiments.

WCR neonate extracts were prepared in PBST containing protease inhibitors (Sigma) and total protein levels measured by BCA total protein assay (Pierce). Extracts were prepped with 2X LDS buffer (Invitrogen) containing DTT as a reducing agent and heated at 70°C for 5 min. Equal amounts of total protein (30 µg) were loaded onto 10% Bis-Tris gels (Invitrogen) and run in MOPS running buffer at 150 V. Proteins were transferred to a 0.2 µM nitrocellulose membrane (Bio-Rad) at 100 V for one hour in transfer buffer (Invitrogen) containing 20% methanol and blocked overnight at 4°C with PBST containing 2% nonfat dry milk. Blots were probed with rabbit anti-DvSNF7 IgG at 1 µg/mL at room temp for 1 hour and washed 4×5 minutes in PBST. A goat anti-rabbit-HRP secondary antibody (Thermo, 1∶200,000 dilution) was used to probe blots for one hour at room temp. Blots were washed 4×5 minutes in PBST. Upon incubation with chemiluminescent substrate (Thermo), the signal was detected by exposure to film (Roche). Band densities were measured using a Bio-Rad GS-800 densitometer. Densitometry results were obtained from an average of three replicates per treatment and time-point, loaded on duplicate gels.

Expression levels of DvSNF7 were also measured via a double antibody sandwich (DAS) ELISA. ELISA plates (Thermo) were coated overnight at 4°C with rabbit anti-DvSNF7 polyclonal antibody at 5 µg/mL. Plates were washed four times with PBST between each step, with a BioTek ELX405HT2S plate washer. All incubations were performed for one hour at 37°C. WCR extracts were prepared in PBST and loaded at 15 µg/well (total protein). A biotin labeled anti-DvSNF7 antibody followed by neutravidin-HRP (Thermo) and TMB substrate (Sigma) was used for detection. Reactions were stopped with 3 M phosphoric acid and absorbance measured at 450 nm. Quantification of DvSNF7 levels was performed using standard DvSNF7 protein as reference. DvSNF7 expression levels were measured for three replicates per treatment and time point, loaded on duplicate plates.

### Statistical Analyses

Comparisons of mortality among treatments groups was performed with a one-sided Fisher's Exact Test (p = 0.05). The standard PROBIT model, under PROC PROBIT in SAS (SAS 9.2, SAS Institute Inc., Cary, NC, USA) was used to estimate the LC_50_ values using the OPTC function to correct for control mortality. Significance of concentration-response curves was evaluated with a Chi-square test (p = 0.05). In one case where PROC PROBIT did not provide a 95% confidence interval for a given LC_50_ value, the diet concentrations bracketing the LC_50_ value are provided as an estimate of the 95% confidence intervals. Slopes for concentration response curves were compared with an extra sum-of-squares F-test (p = 0.05) or 95% confidence intervals are reported. For gene expression studies analysis of variance (ANOVA) was run following a complete randomized design and the samples were compared with each other using the mean separation technique which provides the letter grouping based on the pair-wise t test from SAS PROC GLIMMIX at the significance level of alpha  = 0.05 (SAS 9.2, SAS Institute Inc., Cary, NC, USA).

## Supporting Information

File S1 **Details of gene sequences used in the study.**
(DOCX)

Table S1 **Primer sequences used in the study.**
(DOCX)
