# Supplementary material for: Characterizing the Mechanism of Action of Double-Stranded RNA Activity against Western Corn Rootworm (Diabrotica virgifera virgifera LeConte)
Source: PLoS One. 2012 Oct 11;7(10):e47534. doi: 10.1371/journal.pone.0047534 (PMC3469495; doi:10.1371/journal.pone.0047534)
Supplement: File S1 — Details of gene sequences used in the study. (DOCX) [file pone.0047534.s001.docx]

**Supplementary Information**

**S1. Details of gene sequences used in the study**

All sequences are in 5’-3’ orientation

**DvSnf7_240 bp**

GCAAAGAAAAATGCGTCGAAAAATAAAAGAGTTGCACTCCAAGCCCTCAAAAAGAAGAAACGATTGGAAAAGACCCAACTACAAATAGATGGAACCCTTACAACTATTGAAATGCAGAGGGAAGCCCTCGAAGGAGCTAGCACAAATACTGCTGTATTAGATTCTATGAAAAATGCTGCAGATGCCCTTAAGAAAGCTCATAAGAATTTGAATGTAGATGATGTTCACGATATCATGGAT

**GFP**

GCCAGATACCCAGACCACATGAAGCAGCACGACTTCTTCAAGTCTGCCATGCCAGAGGGTTACGTGCAGGAGAGAACCATCTTCTTCAAGGACGACGGTAACTACAAGACCAGAGCCGAGGTGAAGTTCGAGGGTGACACCCTGGTGAACAGAATCGAGCTGAAGGGTATCGACTTCAAGGAGGACGGTAACATCCTGGGTCACAAGCTGGAGTACAACTACAACTCTCACAACGTGTACATCATGGCCGACAAGCAGAAGAACGGTATCAAGGTGAACTTCAAGATCAGACACAACATCGAGGACGGTTCTGTGCAGCTGGCCGACCACTACCAG

**Tubulin**

CCAAGAGAGCTTTCGTCCACTGGTATGTAGGAGAGGGTATGGAAGAAGGTGAATTCTCTGAAGCTCGTGAAGATTTGGCTGCTTTGGAGAAAGATTATGAAGAAGTTGGTATGGACTCCGGAGAAGGTGAGGGTGAAGGAGCTGAA

**240bp filler**

GCCAAAGAGGATACTAACCAAAAGCTGGGCGAGTCAGATGAGGTTCATAATGTTACACGACAGAGAAAGCTCAGTGCCTGAGTGAGATACTTTTGTAGATTAAGTGCATAATGTCAAAGTGAAAAAAAAGGTACATCTAAGATAGGAAGGCGAGAATGAATTATGGAAATATCAATAGGCCCACGTTCCTCCTTGAAGAGCGCTTATATAAATCATTATAACAAGAAGGACAAGGCGAAT

**27mer in 240 bp**

GCCAAAGAGGATACTAACCAAAAGCTGGGCGAGTCAGATGAGGTTCATAATGTTACACGACAGAGAAAGCTCAGTGCCTGAGTGAGATACTTTTGTAGATTAAGTGCTAGATGGAACCCTTACAACTATTGAAAATCTAAGATAGGAAGGCGAGAATGAATTATGGAAATATCAATAGGCCCACGTTCCTCCTTGAAGAGCGCTTATATAAATCATTATAACAAGAAGGACAAGGCGAAT

**27mer in 150 bp**

CATAATGTTACACGACAGAGAAAGCTCAGTGCCTGAGTGAGATACTTTTGTAGATTAAGTGCTAGATGGAACCCTTACAACTATTGAAAATCTAAGATAGGAAGGCGAGAATGAATTATGGAAATATCAATAGGCCCACGTTCCTCCTTG

**27mer in 100 bp**

TCAGTGCCTGAGTGAGATACTTTTGTAGATTAAGTGCTAGATGGAACCCTTACAACTATTGAAAATCTAAGATAGGAAGGCGAGAATGAATTATGGAAAT

**27mer in 90 bp**

GCCTGAGTGAGATACTTTTGTAGATTAAGTGCTAGATGGAACCCTTACAACTATTGAAAATCTAAGATAGGAAGGCGAGAATGAATTATG

**27mer in 80 bp**

AGTGAGATACTTTTGTAGATTAAGTGCTAGATGGAACCCTTACAACTATTGAAAATCTAAGATAGGAAGGCGAGAATGAA

**27mer in 70 bp**

GATACTTTTGTAGATTAAGTGCTAGATGGAACCCTTACAACTATTGAAAATCTAAGATAGGAAGGCGAGA

**27mer in 60 bp**

TTTTGTAGATTAAGTGCTAGATGGAACCCTTACAACTATTGAAAATCTAAGATAGGAAGG

**27mer in 50 bp**

TAGATTAAGTGCTAGATGGAACCCTTACAACTATTGAAAATCTAAGATAG

**27mer in 40 bp**

TAAGTGCTAGATGGAACCCTTACAACTATTGAAAATCTAA

**27 bp**

TAGATGGAACCCTTACAACTATTGAAA

**21 bp**

21.1 TAGATGGAACCCTTACAACTA

21.2 -AGATGGAACCCTTACAACTAT

21.3 --GATGGAACCCTTACAACTATT

21.4 ---ATGGAACCCTTACAACTATTG

21.5 ----TGGAACCCTTACAACTATTGA

21.6 -----GGAACCCTTACAACTATTGAA

21.7 ------GAACCCTTACAACTATTGAAA
