# Supplementary material for: Characterizing the Mechanism of Action of Double-Stranded RNA Activity against Western Corn Rootworm (Diabrotica virgifera virgifera LeConte)
Source: PLoS One. 2012 Oct 11;7(10):e47534. doi: 10.1371/journal.pone.0047534 (PMC3469495; doi:10.1371/journal.pone.0047534)
Supplement: Table S1 — Primer sequences used in the study. (DOCX) [file pone.0047534.s002.docx]

**Table S1. Primer sequences used in the study**

| **Name** | **Primers (5’-3’)** |
| --- | --- |
| For real-time RT-PCR | |
| DvSnf7 | For:CCGACGATCTGGATGACGA  Rev: TTACGAGGCCCAGGCTTCC |
| Tubulin | For:CCAAGAGAGCTTTCGTCCAC  Rev: TTCAGCTCCTTCACCCTCAC |
| For dsRNA synthesis | |
| DvSnf7 | For1: GCAAAGAAAAATGCGTCG  Rev2: ATCCATGATATCGTGAACATC  For2: taatacgactcactatagggGCAAAGAAAAATGCGTCG  Rev1: taatacgactcactatagggATCCATGATATCGTGAACATC |
| GFP | For: taatacgactcactatagggGCCAGATACCCAGACCACAT  Rev: taatacgactcactatagggATTGGGGTGTTCTGCTGGTA |
